# Prognostic factors for early functional outcome in patients with epidural hematoma

**DOI:** 10.1016/j.bas.2026.106666

**Published:** 2026-09-05

**Authors:** Petra A. Mercea, Brad M. Harrington, Iain S. Walker, Ruan Grobler, Matthias Tomschik, Mohammed Zahier Ebrahim, David Roytowski, Armin Gretschel, Adriaan J. Vlok

**Affiliations:** a Department of Neurosurgery, Tygerberg Academic Hospital, Stellenbosch University, Francie van Zijl Drive, Cape Town, 7505, South Africa; b Department of Neurosurgery, Christian Doppler Clinic, Paracelsus Medical University, Ignaz-Harrer-Strasse 79, Salzburg, 5020, Austria

**Keywords:** Epidural hematoma, Traumatic brain injury, Prognostic factors, Time-to-Surgery, Resource limited countries, Early functional outcome

## Abstract

**Objective:**

Epidural hematoma (EDH) is an important manifestation of traumatic brain injury (TBI), yet most evidence originates from high-income countries. This study evaluated prognostic factors and the association between surgical timing and early functional outcome in EDH patients treated in a resource-limited setting.

**Methods:**

In this retrospective single-center study, adult EDH patients admitted to a tertiary hospital between 2011 and 2022 were analyzed. Clinical and radiological data were retrospectively collected. Early functional outcome was assessed using the Glasgow Outcome Scale Extended (GOSE) at discharge. Factors associated with unfavorable outcome were evaluated using uni- and multivariable logistic regression.

**Results:**

Among 225 patients, interpersonal violence (62.2%) was the predominant injury mechanism. Most patients presented with mild TBI (51.1%), and 85.3% achieved a favorable outcome at discharge. Increasing age, abnormal pupillary status, and greater TBI severity were associated with unfavorable outcome in univariable analysis. In multivariable models adjusting for age and TBI severity, neither injury-to-surgery nor admission-to-surgery time was independently associated with outcome. TBI severity remained significantly associated with outcome, with severe TBI conferring approximately fivefold higher odds of unfavorable outcome compared with mild TBI.

**Conclusion:**

TBI severity was the principal independent clinical factor associated with early functional outcome, whereas surgical timing was not independently associated with outcome after adjustment. Crude differences in surgical timing should therefore be interpreted in the context of severity-driven clinical prioritization rather than as evidence that surgical delay is beneficial.

## Highlights

• Severe TBI patients underwent earlier surgical evacuation of EDH.
• Greater TBI severity was associated with increased radiological mass effect.
• Severe TBI independently predicted unfavorable early functional outcome.
• Surgical timing was not independently associated with early functional outcome.
• Interpersonal violence was the leading mechanism of EDH injury in this South African cohort.

## 1 Introduction

Epidural hematomas (EDH) occur as a result of traumatic brain injury (TBI) with the latter having an incidence of 27 million new cases per year (Yang et al., 2021; GTBIaSCI, 2019). They have a relatively low incidence (<10%) among TBI patients with the most common causes being road traffic accidents (15-74%), falls (11-33%), and interpersonal violence (up to 20%). Most epidemiological data about prognostic factors in EDH patients come from high-income countries (HIC) and only limited data from low- and middle-income countries (LMIC) are available (Basamh et al., 2016; Peres et al., 2018; Rahimi et al., 2022; Rohadi Muhammad Rosyidi et al., 2019). EDH typically occur due to low-energy mechanisms delivered focally, and less overall brain injury, often presenting with a favorable Glasgow Coma Scale (GCS) (Rohadi Muhammad Rosyidi et al., 2019; Araujo et al., 2012). Hematoma increase can lead to rapid and dramatic clinical deterioration, making EDH a dangerous entity and a good presenting GCS can provide false reassurance. The initial GCS has a measurable impact on prognosis with a high predictive value (Rohadi Muhammad Rosyidi et al., 2019; Erşahin et al., 1993; Gurer et al., 2017; Gutowski et al., 2018; Heinzelmann et al., 1996; Kulesza et al., 2020; Leitgeb et al., 2013; Lobato et al., 1988; Marmarou et al., 2007). The influence of time interval from trauma to EDH evacuation on patient outcomes remains unclear. Intuitively, it should impact the outcome, yet this has been inconsistent in the literature, as studies are limited by small cohorts and variability in emergency care access. Many of the studies published originate from HIC where rapid access to emergency care is readily available, thus, the prognostication of the influence of time intervals on outcome might not be universally applicable (Rohadi Muhammad Rosyidi et al., 2019; Gutowski et al., 2018; Leitgeb et al., 2013; Lobato et al., 1988; Rivas et al., 1988; Rehman et al., 2010).

This study aimed to analyse factors influencing early functional outcome in patients with EDH, focusing on time interval to surgery and identify indicators for early surgery in a large patient cohort from a country where limited resources impede neurosurgical management.

## 2 Methods

### 2.1 Study design and participants

This single-center retrospective study analyzed EDH patients admitted to a tertiary hospital from January 2011 to January 2022. At this institution approximately 600 isolated TBI cases are managed annually with the majority resulting from road traffic injuries and interpersonal violence. Inclusion criteria were adult patients (≥18 years) with traumatic EDH and sufficiently complete clinical and radiological records. The study followed the Declaration of Helsinki, is consistent with Good Clinical Practice, and received ethical and institutional approval (N21/10/113). No written informed consent was required, as we analyzed pre-existing data and no additional risk was imposed on patients, in accordance with institutional and ethical guidelines.

### 2.2 Data sources and variables

Participants were identified by admission ICD-10 codes and electronically archived clinical records were retrieved. Radiological data, collected from the hospital's digital Picture Archiving and Communication System and processed by a single observer via OsiriX MD (version 11.0, Pixmeo SARL, Switzerland). Radiological measurements of clot volume (cm^3^) were assessed using the volumetric measurement tool, while midlineshift (mm) (MLS) and clot thickness (mm) were measured manually using the ruler function. Data collected included demographics, mechanism of injury, clinical findings (GCS, pupillary status ), timelines to interventions, and outcome data. TBI severity was determined according to the TBI score and classified into mild TBI with GCS 13-15, moderate TBI with GCS 9-12, and severe TBI with GCS <9. Time of injury was obtained from emergency service records, while admission, surgery, and discharge times were collected from clinical records. Treatment of EDH was recorded as operative evacuation of hematoma or conservative treatment; details regarding the specific operative technique were not systematically available for this retrospective analysis. Early functional outcome was assessed using the Glasgow Outcome Scale–Extended (GOSE) at hospital discharge. Length of hospital stay was calculated from admission to discharge. In the present resource-constrained setting, standardized long-term follow-up was not routinely available, and in many cases postoperative care and subsequent appointments were generally continued at local community hospitals. Therefore, discharge GOSE was used as a measure of early functional outcome.

### 2.3 Statistical methods and word processing

As this was a retrospective study, no a priori sample size calculation was performed, and all consecutive eligible patients were included. Continuous variables are presented as mean ± standard deviation (SD) or median with interquartile range (IQR; 25th–75th percentile), according to their distribution. Between-group comparisons were performed using Student's *t*-test or the Mann–Whitney *U* test, as appropriate. Comparisons between more than two non-normally distributed groups were performed using the Kruskal–Wallis test with Dunn–Bonferroni-adjusted post hoc comparisons. Categorical variables were compared using the chi-square or Fisher's exact test.

Binary logistic regression was used to identify factors associated with unfavorable outcome. Univariable analyses were performed for individual candidate predictors, with odds ratios (ORs) and 95% confidence intervals (CIs) reported. To assess the independent association between surgical timing and outcome, multivariable models included age, TBI severity at admission, and time-to-surgery, based on clinical relevance and the prespecified hypothesis of severity-related confounding. TBI severity was modeled categorically with mild TBI as the reference category, whereas age and time-to-surgery were entered as continuous variables. Admission-to-surgery and injury-to-surgery intervals were evaluated in separate models. To reduce the risk of overfitting, multivariable models were kept parsimonious, and the number of predictor parameters was restricted relative to the number of outcome events. An exploratory subgroup analysis was performed among patients with severe TBI to compare clinical, radiological, and timing characteristics according to outcome. Missing data were handled by complete-case analysis. All tests were two-sided, with *p* < .05 considered statistically significant. Given the exploratory study design, no general correction for multiple comparisons was applied. Analyses were performed using SPSS (version 31.0.0.0), IBM Corp., Armonk, NY, USA) and R version 4.2.2 (R Foundation for Statistical Computing, Vienna, Austria).

## 3 Results

Altogether, our study cohort comprised 225 patient suffering from traumatic EDH who were admitted to the department of neurosurgery between January 2011 and January 2022. Preoperative CT scans were available for 203 cases (90.2%). Missing preoperative CT scans were primarily due to the transfer of patients from local primary hospitals to the neurosurgical department and as this was a retrospective study, not all external radiological data could be retrieved.

### 3.1 Patient characteristics and early functional outcome

Our cohort had a strong male dominance (n = 218, 96.9%) with a median age of 28 years (IQR: 23 – 35; range 18-83 years), with only one patient (0.4%) aged ≥ 65 years. The most common mechanism of injury was interpersonal violence (assault, without stabbing) (n = 140, 62.2%), followed by road traffic accidents (n = 42, 18.7%) and falls (n = 21, 9.3%). In 4 (1.8%) cases EDH resulted due to stabbing and in 5 (2.2%) cases due to sports injuries. Admission TBI severity was mild in 51.1% (n = 115), moderate in 21.3% (n = 48), and severe in 27.6% (n = 62) of patients. An abnormal pupillary status was recorded in 41 (18.2%) patients. Isolated head injury was present in 75.1% (n = 169) patients and additional extracranial injuries identified in 55 (24.4%) patients. 177 patients (78.7%) had surgical evacuation of the EDH. Length-of-stay data were available for 214 patients (95.1%), with a median hospital stay of 4 days (IQR: 2–8). At discharge 192 (85.3%) of patients had a favorable outcome, and 33 (14.7%) an unfavorable outcome. Most patients (n = 132, 58.7%) had a GOSE of 8. The mortality rate was 4.9% (n = 11). Demographic data and outcomes are presented in Table 1.

**Table 1:** Patient characteristics and outcome.

Parameters	n	*(%)*
No. of patients	225	*(100%)*
Sex		
Female	7	*(3.1%)*
Male	218	*(96.9%)*
Median age in years^a^	28	*(IQR: 23 – 35)*

Mechanism of injury		
Assault	140	*(62.2%)*
Road traffic accident	42	*(18.7%)*
Fall	21	*(9.3%)*
Sports injury	5	*(2.2%)*
Stabbing	4	*(1.8%)*
Others	13	*(5.8%)*
TBI score on admission		
Mild	115	*(51.1%)*
Moderate	48	*(21.3%)*
Severe	62	*(27.6%)*
Pupils		
Equal and reactive	184	*(81.8%)*
Abnormal status	41	*(18.2%)*
Isolated head injury^a^	169	*(75.1%)*
Extracranial injury	55	*(24.4%)*
Surgical EDH evacuation		
Yes	177	*(78.7%)*
No	48	*(21.3%)*
Dichotomized GOSE^b^ on discharge		
unfavorable (1-5)	33	*(14.7%)*
favorable (6-8)	192	*(85.3%)*
Mortality	11	*(4.9%)*

**Time intervals (h)**	**Median**	***IQR***

injury-to-admission^c^	8.5	*(3-37.8)*
admission-to-surgery^d^	13	*(7-25)*
injury-to-surgery^e^	32	*(17-68.5)*

TBI = Traumatic brain injury; EDH = Epidural hematomas, GOSE = Glasgow Outcome Scale Extended; IQR = Interquartile range.

a Data missing in 1 patient.

b GOSE: (1) Dead, (2) Vegetative state (Absence of awareness of self and environment), (3) Lower severe disability (Needs full assistance in ADL), (4) Upper severe disability (Needs partial assistance in ADL), (5) Lower moderate disability (Independent, but cannot resume work/school or all previous activities), (6) Upper moderate disability (Some disability exists, but can partly resume work or previous activities), (7) Lower good recovery (Minor physical or mental deficits that affect daily life), (8) Full recovery or minor symptoms that do not affect daily life.

c n = 224; data missing in 1 patient.

d n = 174; data missing in 3 of all operated patients (n = 177).

e n = 176; data missing in 1 patient of all operated patients (n = 177).

### 3.2 Radiological characteristics

The EDH was most commonly located over the parietal lobe (n = 77, 37.9%), followed by the frontal (n = 72, 35.5%), temporal (n = 35, 17.2%), and occipital lobes (n = 6, 3.0%), while 13 EDHs (6.4%) were located in the posterior fossa. Other intracranial lesions were contre-coup lesions (n = 76, 37.4%), traumatic subarachnoid hemorrhage (n = 44, 21.7%), subdural hematoma (n = 43, 21.2%), intracerebral hemorrhage (n = 19, 9.4%), intraventricular hemorrhage (n = 5, 2.5%), and ipsilateral (n = 88, 43.4%) or contralateral (n = 67, 33%) hemorrhagic contusions. The median MLS was 4.6 mm (IQR: 1.5-8.5), median clot thickness was 23 mm (IQR: 15.5 – 28.6) and median clot volume was 46.3 cm^3^ (IQR: 20.4 – 78.4). Radiological characteristics are presented in Table 2.

**Table 2:** Radiological characteristics.

Parameters	n	*(%)*
Total No. of patients	225	*(100%)*
Complete radiological data	203	*(90.2%)*

EDH location		
parietal	77	*(37.9%)*
frontal	72	*(35.5%)*
temporal	35	*(17.2%)*
posterior fossa	13	*(6.4%)*
occipital	6	*(3.0%)*

Other intracranial lesions		
contre-coup	76	*(37.4%)*
traumatic SAH	44	*(21.7%)*
SDH	43	*(21.2%)*
ICH	19	*(9.4%)*
IVH	5	*(2.5%)*

Hemorrhagic contusions		
ipsilateral	88	*(43.4%)*
contralateral	67	*(33.0%)*

Midlineshift (mm)		
median	4.6	*(IQR: 1.5-8.5)*
Clot thickness^a^ (mm)		
median	23	*(IQR: 15.5-28.6)*
Clot volume (cm^3^)		
median	46.3	*(IQR: 20.4-78.4)*

EDH = Epidural hematoma; SAH = subarachnoid hemorrhage; SDH = subdural hematoma; ICH = intracerebral hemorrhage; IVH = intraventricular hemorrhage.

a Data missing in 6 patients.

### 3.3 Time intervals and early functional outcome

The median time from injury to admission at tertiary care was 8.5 h (IQR: 3–37.8), while the median admission-to-surgery interval was 13 h (IQR: 7–25), resulting in a median overall injury-to-surgery time of 32 h (IQR: 17–68.5). When stratified by outcome, patients with an unfavorable outcome had a median injury-to-admission time of 9 h (IQR: 3–32.5), a median admission-to-surgery time of 8.5 h (IQR: 4.8–19.3), and an overall injury-to-surgery time of 19 h (IQR: 10–61). In comparison, patients with a favorable outcome had corresponding median intervals of 8 h (IQR: 3–38) injury-to-admission interval, 13 h (IQR: 8–27.5) admission-to-surgery interval, and 35 h (IQR: 18–70) injury-to-surgery interval. Injury-to-admission time did not differ significantly between outcome groups, whereas both admission-to-surgery (p = .040) and injury-to-surgery (p = .038**)** intervals were significantly longer in patients with favorable outcomes (Table 3).

**Table 3:** Group comparison of clinical and radiological characteristics and time intervals between patients with favorable and unfavorable outcomes.

Parameters	Unfavorable outcome^a^		Favorable outcome^a^		*p-value*
**Patients (n=225)**	**33**	*(14.7%)*	**192**	*(85.3%)*	

**Clinical Factors**					
Median age, in years *(IQR*^d^*)*	32.5 *(25.3-39)*		*28 (23-33.8)*		***.017***
Pupils equal and reactive	22	*(66.7%)*	162	*(84.4%)*	***.029***
Abnormal pupillary status	11	*(33.3%)*	30	*(15.6%)*	
TBI severity					***.001***
Mild	6	*(18.2%)*	109	*(56.8%)*	
Moderate	9	*(27.3%)*	39	*(20.3%)*	
Severe	18	*(54.5%)*	44	*(22.9%)*	
Mechanism of injury					*.094*
Interpersonal violence	20	*(60.6%)*	120	*(62.5%)*	
Falls	5	*(15.2%)*	16	*(8.3%)*	
Road traffic accidents	3	*(9.1%)*	39	*(20.3%)*	
Stabbing	0	*(0%)*	4	*(2.1%)*	
Sports injuries	0	*(0%)*	5	*(2.6%)*	
Others	5	*(15.1%)*	8	*(4.2%)*	
Isolated head trauma	26	*(78.8%)*	143	*(74.9%)*	*.816*
Extracranial injuries	7	*(21.2%)*	48	*(25.1%)*	

**Radiological factors**^b^	**Median**	***IQR***	**Median**	***IQR***	
Midline shift (mm)^c^	5.2	*(1.48-8.73)*	4.6	*(1.4-8.45)*	*.739*
Clot thickness (mm)^c^	24.1	*(16.63-28.9)*	22.8	*(15.5-28.6)*	*.754*
Clot volume (cm^3^)^c^	65.0	*(43.1-99)*	46.4	*(20.35-74.45)*	*.624*

**Time intervals (h)**	**Median**	***IQR***	**Median**	***IQR***	
Injury-to-admission^d^	9	*(3-32.5)*	8	*(3-38)*	*.521*
Admission-to-surgery^e^	8.5	*(4.8-19.3)*	13	*(8-27.5)*	***.040***
Injury-to-surgery^6^	19	*(10-61)*	35	*(18-70)*	***.038***

TBI = traumatic brain injury, GOSE = Glasgow outcome scale extended, IQR = Interquartile range.

a Unfavorable outcome (GOSE 1-5), favorable outcome (GOSE 6-8).

b Radiological data available in 203 (92.2%) of all 225 patients.

c Mean values are displayed for chi^2^ and Fishers' exact test.

d n = 224; data missing in 1 patient.

e n = 174; data missing in 3 of all operated patients (n = 177).

### 3.4 Comparison of clinical and radiological characteristics according to early functional outcome

**Fig. 1:**
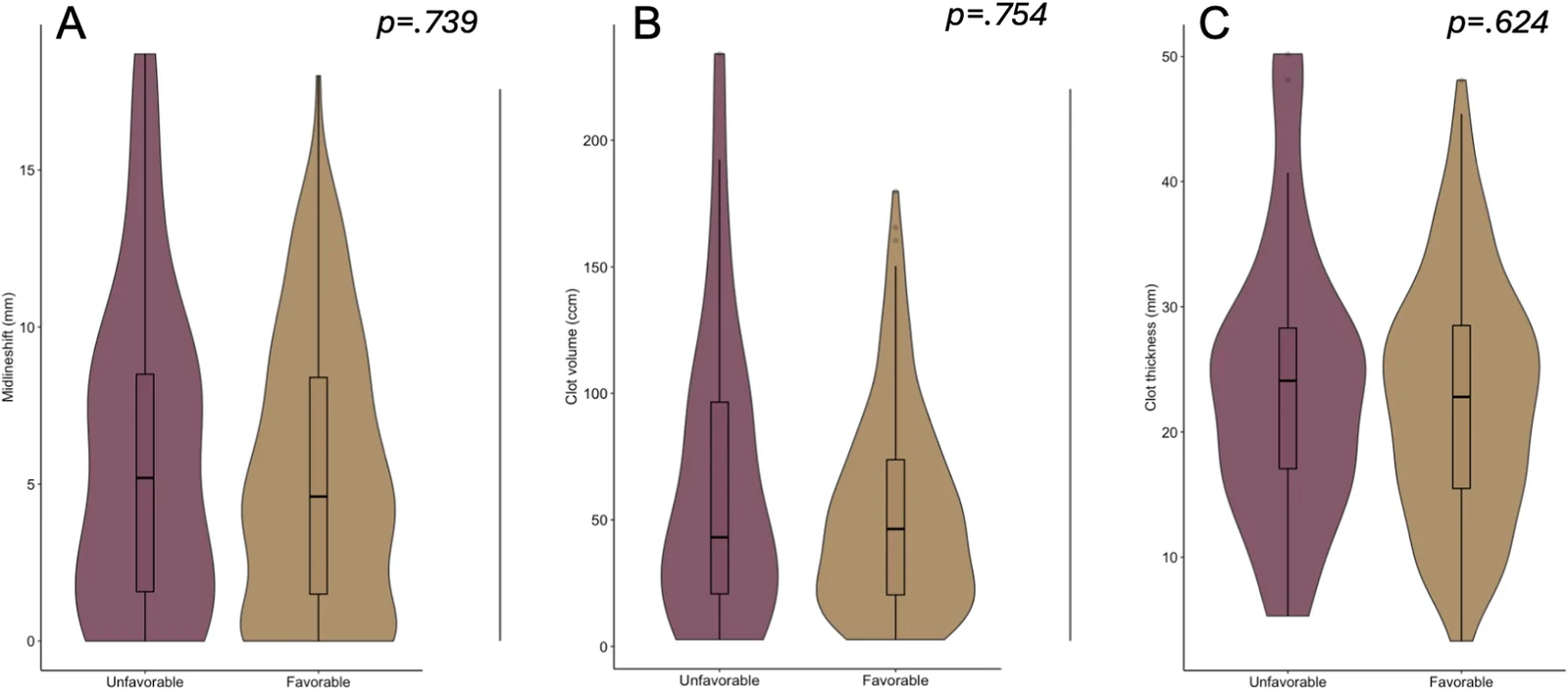
**Comparison of radiological parameters according to early functional outcome.** No significant differences were observed between patients with unfavorable and favorable outcomes in **(A)** midlineshift [median 5.2 mm (IQR: 1.48–8.73) vs. 4.6 mm (IQR: 1.4–8.45), *p* = .739], **(B)** clot thickness [median 24.1 mm (IQR: 16.63–28.9) vs. 22.8 mm (IQR: 15.5–28.6), *p* = .754], or **(C)** clot volume [median 65.0 cm^3^ (IQR: 43.1–99.0) vs. 46.4 cm^3^ (IQR: 20.35–74.45), *p* = .624]. Comparisons were performed using the Mann–Whitney *U* test. IQR = interquartile range.

In group comparison analysis patients with favorable outcomes were significantly (p = .017) younger (median age: 28, IQR: 23-33.8) than patients with unfavorable outcome (median age: 32.5 years, IQR: 25.3-39). An abnormal pupillary status was significantly associated with worse outcome (p = .029). Patients with severe TBI fared worse, with 54.5% experiencing unfavorable outcomes, compared to moderate TBI with 27.3%, and mild TBI with only 18.2% having unfavorable outcomes (p = .001). The mechanism of injury and the presence of extracranial injuries showed no significant association with patients' outcomes. Radiological factors such as MLS, clot thickness and volume did not significantly influence patients’ outcome (Table 3). Also, EDH location was not significantly associated with early functional outcome (p = .852). In the exploratory subgroup analysis restricted to patients with severe TBI (n = 62), no significant differences in age, pupillary status, MLS, clot thickness, clot volume, or time intervals (injury-to-admission, admission-to-surgery, injury-to-surgery) were observed between patients with favorable and unfavorable outcomes (Supplementary Table 1). Association of radiological factors and outcome are depicted in Fig. 1.

### 3.5 Clinical and radiological characteristics according to treatment strategy and surgical timing

**Fig. 2:**
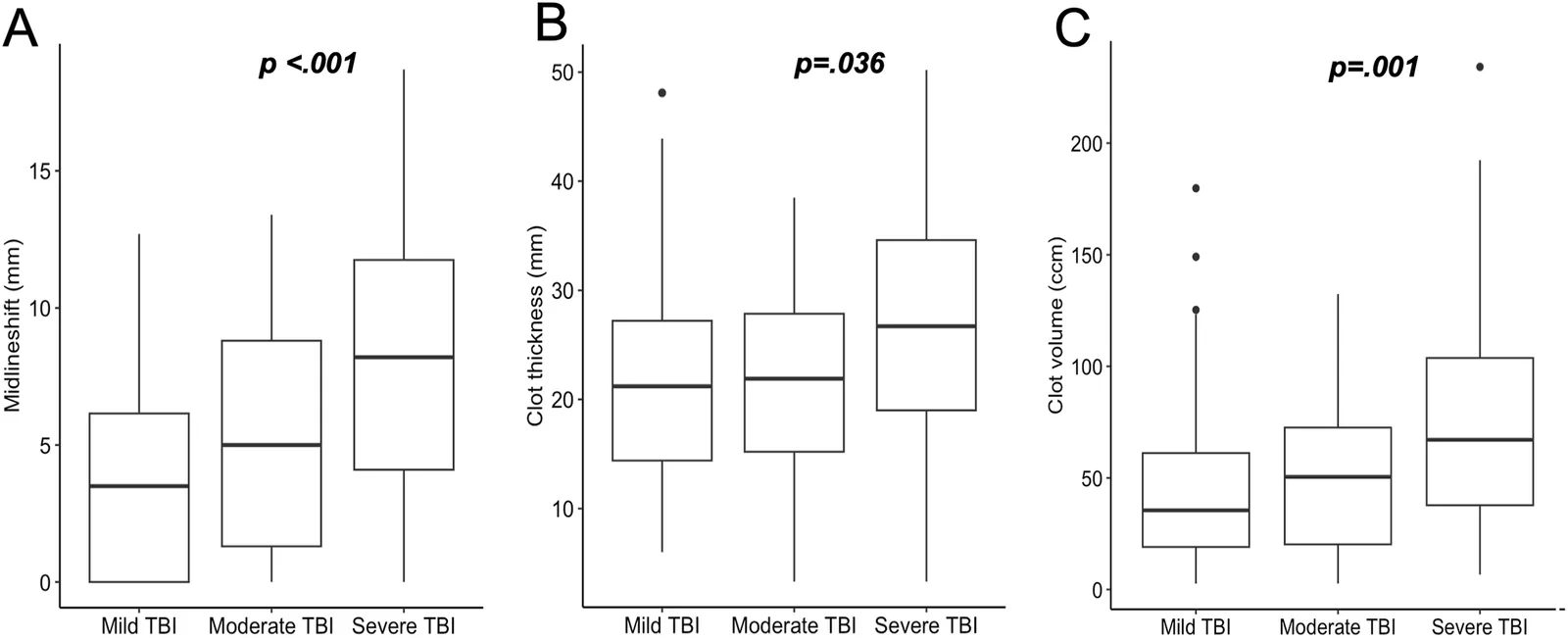
**Radiological characteristics according to traumatic brain injury severity.** Radiological mass effect increased with TBI severity. **(A)** Median midlineshift was 3.5 mm in patients with mild TBI, 5.0 mm in those with moderate TBI, and 8.2 mm in those with severe TBI (***p*<.001**). **(B)** Median clot thickness was 21.2 mm, 21.9 mm, and 26.7 mm in patients with mild, moderate, and severe TBI, respectively (***p*=.036**). **(C)** Median clot volume was 35.5 cm^3^, 50.5 cm^3^, and 67.1 cm^3^ in patients with mild, moderate, and severe TBI, respectively (***p*=.001**). Overall group differences were assessed using the Kruskal–Wallis test. TBI= traumatic brain injury.

**Fig. 3:**
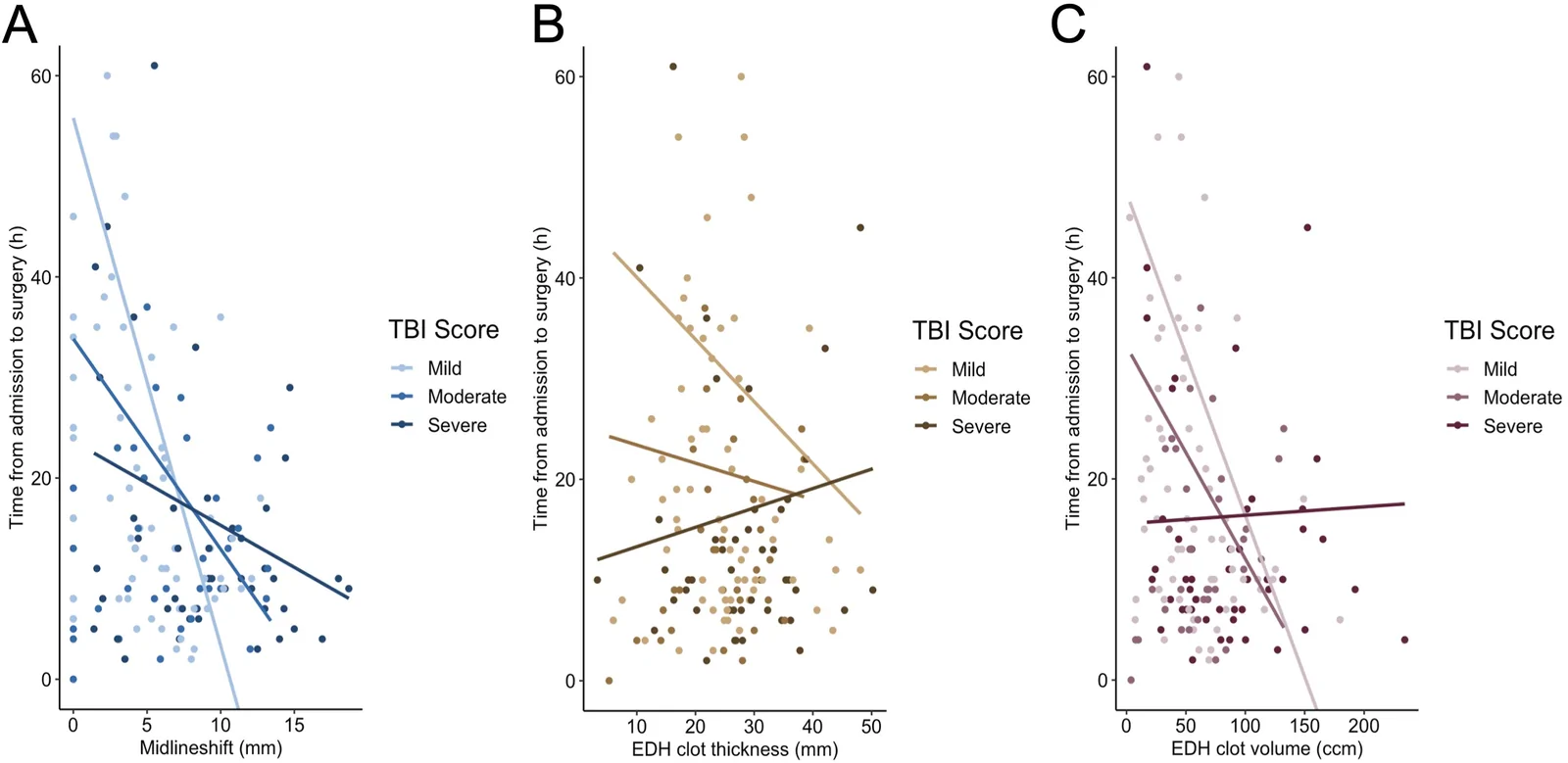
**Relationship between radiological mass effect and timing of surgical evacuation according to traumatic brain injury severity.** Surgical timing according to **(A)** midlineshift, **(B)** clot thickness, and **(C)** clot volume is shown across mild, moderate, and severe TBI groups. Greater radiological mass effect was generally observed in patients undergoing earlier surgical evacuation, particularly among patients with mild and moderate TBI. Patients with severe TBI underwent earlier surgical intervention across a broad range of radiological measurements, consistent with prioritization according to neurological injury severity. MLS = midlineshift; TBI = traumatic brain injury.

Midlineshift, clot thickness, and clot volume differed significantly according to treatment strategy. Patients undergoing surgery demonstrated a greater median MLS (6.15 mm, IQR: 3.08–9.3 vs. 0 mm, IQR 0–3), clot thickness (25.5 mm, IQR: 19.1–30.18 vs. 12.1 mm, IQR: 8.7–17.7), and clot volume (56.0 cm^3^, IQR: 34.13–88.65 vs. 12.8 cm^3^, IQR: 6.9–21.8) compared with patients managed conservatively (all *p* < .001) (Table 5). In contrast, EDH location and TBI severity did not differ significantly between treatment groups (*p* = .344 and *p* = .124, respectively). Time to admission and surgery decreased with increasing TBI severity. Patients with severe TBI had shorter injury-to-admission and admission-to-surgery intervals than patients with mild TBI (*p* < .001 and *p* = .003, respectively). Greater TBI severity was also associated with increased radiological mass effect, with significantly greater MLS, clot thickness, and clot volume observed in patients with severe compared with mild TBI (Fig. 2A–C). Greater radiological mass effect was generally observed in patients undergoing earlier surgical evacuation, particularly among those with mild and moderate TBI (Fig. 3).

### 3.6 Univariable and multivariable logistic regression analyses of unfavorable outcome

In univariable logistic regression analysis, increasing age was associated with higher odds of an unfavorable outcome (OR 1.043 per year, 95% CI 1.008–1.079; *p* = .016). Abnormal pupillary status was likewise associated with unfavorable outcome compared with PEARL (OR 2.70, 95% CI 1.19–6.14; *p* = .018). TBI severity was significantly associated with outcome overall (*p* < .001); compared with mild TBI, both moderate (OR 4.19, 95% CI 1.40–12.54; *p* = .010) and severe TBI (OR 7.43, 95% CI 2.77–19.96; *p* < .001) were associated with increased odds of an unfavorable outcome. Neither injury-to-surgery time (OR 0.990 per hour, 95% CI 0.977–1.003; *p* = .116) nor admission-to-surgery time (OR 0.972 per hour, 95% CI 0.938–1.007; *p* = .111) was significantly associated with outcome in univariable logistic regression.

Given the observed unadjusted differences in surgical timing according to outcome and TBI severity, surgical timing was subsequently evaluated after adjustment for age and TBI severity. Among the 225 patients, 33 experienced an unfavorable outcome. Each multivariable model comprised four predictor parameters, corresponding to approximately 8.3 events per parameter. In the model including injury-to-surgery time, TBI severity remained significantly associated with outcome overall (*p* = .027), with severe TBI independently associated with increased odds of an unfavorable outcome compared with mild TBI (aOR 5.22, 95% CI 1.56–17.45; *p* = .007). Neither age (aOR 1.038, 95% CI 0.988–1.090; *p* = .143) nor injury-to-surgery time (aOR 0.997 per hour, 95% CI 0.987–1.008; *p* = .617) was independently associated with outcome. Similar findings were observed in the model including admission-to-surgery time: TBI severity remained significant overall (*p* = .019), with severe TBI independently associated with unfavorable outcome (aOR 5.19, 95% CI 1.65–16.37; *p* = .005), whereas age (*p* = .160) and admission-to-surgery time (aOR 0.984 per hour, 95% CI 0.954–1.016; *p* = .327) were not independently associated with outcome (Table 4).

**Table 4:** Univariable and multivariable logistic regression analyses of unfavorable outcome.

Univariable analysis			
**Parameters**	**Unadjusted OR**	**95% CI**	***p-value***

Age, per year	1.043	1.008 – 1.079	***.016***
Abnormal pupillary status (vs. PEARL)	2.700	1.187 – 6.142	***.018***
TBI severity overall	-	-	***< .001***
Moderate vs. mild TBI	4.192	1.401 – 12.542	***.010***
Severe vs. mild TBI	7.432	2.767 – 19.961	***< .001***
Injury to surgery, per hour	0.990	0.977 – 1.003	.*116*
Admission to surgery, per hour	0.972	0.938 – 1.007	*.111*

**Multivariable analysis**	**Adjusted OR**	**95% CI**	*p-value*

**Model 1: Injury-to-surgery**			
Age, per year	1.038	0.988–1.090	*.143*
TBI severity, overall	—	—	***.027***
Moderate vs. mild	2.845	0.773–10.466	*.116*
Severe vs. mild	5.221	1.562–17.449	***.007***
Injury-to-surgery, per hour	0.997	0.987–1.008	.*617*

**Model 2: Admission-to-surgery**			
Age, per year	**1.036**	0.986-1.088	*.160*
TBI severity, overall	—	—	***.019***
Moderate vs. mild	**3*.205***	0.923-11.127	.*067*
Severe vs. mild	*5*.193	1.647-16.369	***.005***
Admission-to-surgery, per hour	0.984	0.954–1.016	*.327*

OR = odds ratio; CI = confidence interval; TBI = traumatic brain injury; PEARL = pupils equal and reactive to light. Mild TBI and PEARL served as reference categories. Model 1 included age, TBI severity, and injury-to-surgery time; Model 2 included age, TBI severity, and admission-to-surgery time.

**Table 5:** Comparison of clinical and radiological characteristics between patients undergoing surgical and conservative management.

	Conservative management		Surgery		*p-value*
**Radiological parameters**^a^	**45**	*(22.2)*	**158**	*(77.8)*	

Location of EDH					*.344*
Frontal	21	*(46.7)*	51	*(32.3%)*	
Temporal	6	*(13.3%)*	29	*(18.4%)*	
Parietal	13	*(28.9%)*	64	*(40.5%)*	
Occipital	1	*(2.2%)*	5	*(3.2%)*	
Posterior fossa	4	*(8.9%)*	9	*(5.7%)*	

	**Median**	***IQR***	**Median**	***IQR***	
Midlineshift (mm)	0	(0-3)	6.15	(3.08-9.3)	***< .001***
Clot thickness (mm)	12.1	(8.7-17.7)	25.5	(19.1-30.18)	***< .001***
Clot volume (cm^3^)	12.8	(6.9-21.8)	56.0	(34.13-88.65)	***< .001***


**Clinical parameters**^b^	**48**	*(21.3)*	**177**	*(78.7)*	

TBI severity					*.124*
Mild	30	*(62.5%)*	85	*(48.0%)*	
Moderate	10	*(20.8%)*	38	*(21.5%)*	
Severe	8	*(16.7%)*	54	*(30.5%)*	

EDH = Epidural hematoma; TBI = Traumatic brain injury, IQR = Interquartile range

a Complete radiological data available in 203 patients (90,2%).

b Complete clinical data available in 225 patients (100%).

Thus, despite the unadjusted differences in surgical timing between outcome groups, neither injury-to-surgery nor admission-to-surgery time was independently associated with unfavorable outcome after adjustment for age and TBI severity.

## 4 Discussion

This study presents a comprehensive analysis on the prognostic factors in a large cohort of patients suffering from traumatic EDH, including surgical timing and its association with early functional outcome.

In our study of 225 EDH patients, 78.7% underwent surgery, while 21.3% were treated conservatively. In comparison, Basamh et al. reported that only 35.9% of their EDH patients underwent surgery, and Sullivan et al. found that 63.5% were managed conservatively, with only 10% later requiring surgery for enlarging EDH (Basamh et al., 2016; Sullivan et al., 1999). Our cohort included a higher proportion of moderate (21.3%) and severe TBI cases (27.6%) compared to Basamh et al. (14.4%), likely due to serving a large population of about five million as the sole neurosurgical service (Basamh et al., 2016).

The 2006 Brain Trauma Foundation guidelines recommend urgent surgical evacuation for patients presenting with acute EDH, a GCS score <9, and anisocoria, with surgery to be performed as rapidly as possible (Jamjoom, 1997). In resource-limited settings, however, timely access to CT imaging, neurosurgical assessment, and operative intervention is frequently constrained and may not be comparable to that achieved in well-resourced health-care systems. Nevertheless, the Global Neurotrauma Outcomes Study, which included 1635 patients across all seven World Bank geographical regions, reported a median interval from injury-to-surgery of 13 h (IQR: 6–32) across the overall cohort (Clark et al., 2022). Among patients undergoing evacuation of a supratentorial EDH or acute subdural hematoma a median time to surgery of 11 h (IQR: 5–25) was observed, which decreased to 8 h (IQR: 4–17) among patients with severe TBI (Clark et al., 2022). Importantly, the study demonstrated progressively increasing delays from injury to surgery across countries ranging from very-high- to low-HDI (human development index) settings, highlighting the influence of socioeconomic and health-care resource disparities on timely neurotrauma care (Clark et al., 2022). In our cohort, the median interval from injury to emergency room admission was 8.5 h (IQR: 3–37.8), while the median time from emergency room admission to surgery was 13 h (IQR: 7–25). The prolonged treatment intervals observed in our setting is likely multifactorial but is primarily attributable to the absence of a dedicated neurosurgical emergency operating theatre, resulting in competition for operative access with other surgical specialties and subsequent delays in triage and surgical scheduling.

Previous studies on the timing of surgery and outcomes in EDH patients have shown inconsistent results (Rohadi Muhammad Rosyidi et al., 2019; Lobato et al., 1988; Jamjoom, 1997). Rosyidi et al. reported higher mortality and morbidity in patients operated on more than 48 h after diagnosis (Rohadi Muhammad Rosyidi et al., 2019). Conversely, another study reported higher mortality among comatose patients undergoing surgery within 6 h or between 6 and 12 h (35% and 33%, respectively) than among those operated later (0% mortality), which the authors attributed to greater neurological and radiological severity among patients requiring early intervention (Lobato et al., 1988). Neurological status at admission has consistently been identified as an important prognostic factor in EDH (Kulesza et al., 2020; Leitgeb et al., 2013). Our results support the prognostic importance of neurological status at admission, with increasing TBI severity associated with worse early functional outcome. In the whole-cohort comparison across TBI severity groups, patients with severe TBI had significantly shorter injury-to-admission and admission-to-surgery intervals than patients with mild TBI, consistent with prioritization according to neurological status and clinical urgency. Severe TBI was also associated with greater radiological mass effect, including greater MLS, clot thickness, and clot volume. Taken together, these results are consistent with severity-driven clinical prioritization and support current emergency triage practices in which patients with severe neurological impairment and significant radiological mass effect undergo expedited surgical intervention (Bullock et al., 2006). Importantly, however, this association should not be interpreted as evidence that earlier surgery itself was associated with worse outcome, but rather as reflecting the greater baseline injury severity of patients requiring urgent intervention.

A retrospective Austrian study including 159 patients with EDH reported that only 7% presented more than 90 min after injury, while surgery was performed within 2 h in 88% of cases (Leitgeb et al., 2013). Similarly, a German study demonstrated improved outcomes when the interval from injury to hospital admission was less than 1 h (Gutowski et al., 2018). These findings reflect highly efficient trauma systems and extensive emergency medical resources in high-income countries (HICs). In contrast, LMICs frequently face substantial limitations in health-care infrastructure and emergency medical services despite bearing a disproportionately high burden of TBI, with incidence rates estimated to be nearly threefold higher than in HICs (Dewan et al., 2018). The Global Neurotrauma Outcomes Study highlighted substantial disparities in emergency neurotrauma care across human development settings, including differences in neurosurgical workforce, CT availability, intracranial pressure monitoring, intensive care capacity, and organized prehospital transport (Clark et al., 2022). Although South Africa is formally classified as a high-HDI country, profound inequalities in access to health care remain. The public health-care sector, serving approximately 84% of the population (∼60 million people), operates under considerable resource constraints, including limited neurosurgical workforce capacity and few specialised neurosurgical centres nationwide. Rapid urbanization and increasing migration into metropolitan areas further increase pressure on already overburdened health-care facilities (Maphumulo and Bhengu, 2019). Consequently, neurotrauma care within the South African public sector may more closely resemble that of middle-HDI settings, and the prolonged treatment intervals observed in our cohort likely reflect limitations in prehospital transport and access to emergency neurosurgical care.

Previous studies have not consistently demonstrated an independent association between shorter time to surgery and improved outcomes (Kulesza et al., 2020; Leitgeb et al., 2013). In our cohort, patients with favorable outcomes had significantly longer median admission-to-surgery intervals than patients with unfavorable outcomes [13 h (IQR: 8–27.5) vs. 8.5 h (IQR: 4.8–19.3), *p* = .040], as well as longer overall injury-to-surgery intervals [35 h (IQR: 18–70) vs. 19 h (IQR: 10–61), *p* = .038]. In contrast, injury-to-admission intervals did not differ significantly between outcome groups. Importantly, however, neither admission-to-surgery nor injury-to-surgery time was independently associated with unfavorable outcome after adjustment for age and TBI severity. The apparent inverse relationship between surgical delay and outcome in the unadjusted comparison is therefore most consistent with confounding by indication, reflecting severity-driven clinical prioritization: patients presenting with greater neurological impairment required more urgent surgery and simultaneously carried a higher baseline risk of unfavorable outcome. In contrast, neurologically stable patients were more likely to undergo surgery after longer intervals and generally had a more favorable baseline prognosis. This distinction is particularly important in EDH, which represents a potentially reversible intracranial mass lesion: timely evacuation in patients with neurological deterioration or significant mass effect may prevent irreversible secondary brain injury and can allow substantial neurological recovery even in critically ill patients. Accordingly, our findings should not be interpreted as evidence that delayed EDH evacuation is beneficial or that timely surgical intervention is unimportant.

The Brain Trauma Foundation guidelines recommend conservative treatment for EDH <30 cm^3^, <15 mm thickness, <5 mm MLS, and GCS >8 without focal deficits (Bullock et al., 2006). An MLS <10 mm has also been associated with better outcomes, although this finding is based on relatively small cohorts (Kulesza et al., 2020). In our study, MLS, clot thickness, and clot volume were not significantly associated with early functional outcome. Notably, frontal and parietal EDHs predominated in our cohort, differing from the predominantly temporal or temporoparietal distribution classically described in adult traumatic EDH, which is frequently associated with middle meningeal artery injury (Tsiouris et al., 2024). This distinct anatomical distribution may partly reflect differences in injury mechanisms, particularly the high prevalence of interpersonal violence in our cohort, although this association was not specifically evaluated. EDH location itself was not significantly associated with early functional outcome. Nevertheless, greater radiological mass effect was associated with more severe TBI and earlier surgical intervention. Greater clot thickness and volume were also associated with earlier surgery among patients with mild and moderate TBI. These findings suggest that radiological mass effect, together with neurological status, was associated with surgical prioritization rather than serving as an independent determinant of outcome. The median clot thickness and clot volume in our cohort exceeded the thresholds commonly used to guide conservative management, reflecting a substantial radiological burden of EDH in this population. Overall, our findings support an integrated approach to surgical decision-making that considers both neurological status and radiological mass effect. Patients with unfavorable outcomes more frequently presented with more severe TBI and pupillary abnormalities, potentially reflecting greater initial injury severity, raised intracranial pressure, and impending transtentorial herniation.

In our multivariable analyses, TBI severity remained significantly associated with early functional outcome, with severe TBI conferring approximately fivefold higher odds of an unfavorable outcome compared with mild TBI. Moderate TBI did not remain statistically significant after adjustment. Increasing age and abnormal pupillary status were associated with unfavorable outcome in univariable analysis. In the multivariable timing models, age did not retain statistical significance, while pupillary status was not included to maintain model parsimony given the limited number of unfavorable events. These findings reinforce the prognostic importance of initial neurological injury severity in patients with EDH (Kulesza et al., 2020; Leitgeb et al., 2013; Marmarou et al., 2007). The predominance of younger patients in our cohort likely reflects the local epidemiology of trauma and the high burden of interpersonal violence among young men (Grobler et al., 2024; Harrington et al., 2021). Only one patient (0.4%) was aged ≥65 years, precluding a meaningful subgroup analysis of geriatric EDH.

Rahimi et al. identified MVA as the most common mechanism of EDH (47.7%), with a higher proportion reported in LMICs (57.1%) than in HICs (44.1%) (Rahimi et al., 2022), while other studies have frequently reported falls (Basamh et al., 2016; Rohadi Muhammad Rosyidi et al., 2019; Leitgeb et al., 2013; Jin et al., 2018). In contrast, interpersonal violence predominated in our cohort (62.2%), whereas road traffic accidents and falls accounted for only 18.7% and 9.3%, respectively. This distinct injury profile reflects the substantial burden of interpersonal violence in South Africa and highlights important regional differences in the epidemiology of traumatic EDH. (Index GOC; Index-Numbeo C) The predominance of interpersonal violence may also partly contribute to the distinct anatomical distribution of EDH observed in our cohort, although this association was not specifically evaluated. However, mechanism of injury was not significantly associated with early functional outcome in our cohort, consistent with findings from previous studies (Basamh et al., 2016; Leitgeb et al., 2013; Jin et al., 2018).

## 5 Limitations

The main important limitation of this study is that selection bias cannot be excluded, as only patients who arrived alive at the tertiary referral center were included; severely injured patients may have died before reaching neurosurgical care. The analysis of surgical timing is further susceptible to confounding by indication, as patients with greater neurological severity were preferentially prioritized for earlier intervention, and to potential survivor/immortal-time bias, since patients undergoing later surgery necessarily had to survive long enough to reach delayed intervention. Although surgical timing was not independently associated with outcome after adjustment for age and TBI severity, residual confounding cannot be excluded and causal conclusions regarding the effect of surgical timing cannot be drawn.

The eleven-year study period may have introduced temporal confounding due to changes in imaging accessibility, transport logistics, and trauma care. Furthermore, preoperative CT imaging was unavailable in a subset of patients. Functional outcome was assessed using GOSE at discharge rather than at standardized long-term follow-up and therefore reflects early functional status only. As hospital length of stay varied between patients, GOSE assessment did not accur at a uniform post-injury time point. Standardized long-term follow-up was not routinely available, as subsequent care was commonly transferred to local hospitals. Direct comparison with studies reporting three- or six-month GOSE should consequently be made with caution.

Finally, the limited number of unfavorable outcome events restricted the complexity of the multivariable models and may have affected the stability and precision of the estimates despite a deliberately parsimonious modeling approach. Radiological measurements were performed by a single investigator, and interobserver reliability was not assessed.

## 6 Conclusion

Our study comprises one of the largest cohort of patients with EDH from a resource-limited public healthcare system. The results highlight the prognostic importance of neurological status at admission, with severe TBI independently associated with unfavorable early functional outcome. Although patients with favorable outcomes demonstrated longer intervals from surgical intervals in unadjusted comparisons, neither injury-to-surgery nor admission-to-surgery time was independently associated with outcome after adjustment for age and TBI severity. These findings suggest that the apparent relationship between surgical timing and outcome primarily reflects severity-driven clinical prioritization rather than an effect of surgical delay itself. Radiological parameters, including MLS, clot thickness and clot volume, were not significantly associated with early functional outcome but were associated with TBI severity and surgical prioritization. Overall, our findings emphasize the importance of integrated clinical and radiological assessment for surgical prioritization in EDH, particularly in resource-constrained settings.

## Ethics declaration

Written informed consent to take part in the study and to publish the article has been obtained from all participants or their legal representatives. The privacy rights of participants have been observed.

This study was performed in compliance with relevant laws, regulatory frameworks and guidelines where the research took place. This study was approved by the Health Research Ethics Committee (HREC), Faculty of Medicine and Health Sciences, Stellenbosch University, South Africa. (Approval No. N21/10/113)

## Consent to participate

The requirement for individual informed consent was waived by the ethics committee due to the retrospective nature of the study.

## Ethics approval

The study was approved by the Health Research Ethics Committee (HREC), Faculty of Medicine and Health Sciences, Stellenbosch University, South Africa (reference no. N21/10/113).

## Consent for publication

Not applicable.

## Data availability

The datasets generated and/or analyzed during the current study are available from the corresponding author on reasonable request, subject to institutional and ethical requirements.

## Declaration of AI-assisted technologies in the manuscript preparation process

During the preparation of this work the authors used ChatGPT (OpenAI) to assist with language editing and improvement of manuscript readability. The authors reviewed and edited the content as needed and take full responsibility for the content of the publication.

## Funding

No specific funding was received for this study.

## Declaration of competing interest

The authors declare that they have no known competing financial interests or personal relationships that could have appeared to influence the work reported in this paper.

## References

[bib1] Araujo J.L., Aguiar Udo P., Todeschini A.B., Saade N., Veiga J.C. (2012). Epidemiological analysis of 210 cases of surgically treated traumatic extradural hematoma. Rev. Col. Bras. Cir..

[bib2] Basamh M., Robert A., Lamoureux J., Saluja R.S., Marcoux J. (2016). Epidural Hematoma treated conservatively: when to expect the worst. Can. J. Neurol. Sci..

[bib3] Bullock M.R., Chesnut R., Ghajar J., Gordon D., Hartl R., Newell D.W. (2006). Guidelines for the surgical management of traumatic Brain Injury author group: acknowledgments. Neurosurgery.

[bib4] Clark D., Joannides A., Adeleye A.O., Bajamal A.H., Bashford T., Biluts H. (2022). Casemix, management, and mortality of patients rreseceiving emergency neurosurgery for traumatic brain injury in the Global Neurotrauma Outcomes Study: a prospective observational cohort study. Lancet Neurol..

[bib5] Dewan M.C., Rattani A., Gupta S., Baticulon R.E., Hung Y.C., Punchak M. (2018). Estimating the global incidence of traumatic brain injury. J. Neurosurg..

[bib6] Erşahin Y., Mutluer S., Güzelbag E. (1993). Extradural hematoma: analysis of 146 cases. Childs Nerv. Syst..

[bib7] Grobler R., Geldenhuys E.M., Vlok A.J. (2024). Fairway to fractures: income inequality and violent crime as the driving factors for golf club-related assaults - a case series of 21 compound skull fractures. S. Afr. Med. J..

[bib8] GtbiaSCI Collaborators (2019). Global, regional, and national burden of traumatic brain injury and spinal cord injury, 1990-2016: a systematic analysis for the global Burden of Disease Study 2016. Lancet Neurol..

[bib9] Gurer B., Kertmen H., Yilmaz E.R., Dolgun H., Hasturk A.E., Sekerci Z. (2017). The surgical outcome of traumatic extraaxial hematomas causing brain herniation. Turk Neurosurg.

[bib10] Gutowski P., Meier U., Rohde V., Lemcke J., von der Brelie C. (2018). Clinical outcome of epidural hematoma treated surgically in the era of modern resuscitation and trauma care. World Neurosurg..

[bib11] Harrington B.M., Gretschel A., Lombard C., Lonser R.R., Vlok A.J. (2021). Complications, outcomes, and management strategies of non-missile penetrating head injuries. J. Neurosurg..

[bib12] Heinzelmann M., Platz A., Imhof H.G. (1996). Outcome after acute extradural haematoma, influence of additional injuries and neurological complications in the ICU. Injury.

[bib13] Index GOC. Global score for criminality 2023 [global score for criminality]. Available from: https://ocindex.net.

[bib14] Index-Numbeo C. Crime Index by Country 2024 Mid-Year 2024 [Crime Index by Country 2024 Mid-Year]. Available from: https://www.numbeo.com/crime/rankings_by_country.jsp.

[bib15] Jamjoom A.B. (1997). The difference in the outcome of surgery for traumatic extradural hematoma between patients who are admitted directly to the neurosurgical unit and those referred from another hospital. Neurosurg. Rev..

[bib16] Jin X.Q., Huang Y.W., Yang M.F. (2018). Association between gray-white matter ratio in computed tomography and outcome in patients with extra-axial hematoma. World Neurosurg..

[bib17] Kulesza B., Litak J., Mazurek M., Nogalski A. (2020). Initial factors affecting 6-month outcome of patients undergoing surgery for acute post-traumatic subdural and epidural Hematoma. Folia Med. (Plovdiv).

[bib18] Leitgeb J., Mauritz W., Brazinova A., Majdan M., Wilbacher I. (2013). Outcome after severe brain trauma associated with epidural hematoma. Arch. Orthop. Trauma Surg..

[bib19] Lobato R.D., Rivas J.J., Cordobes F., Alted E., Perez C., Sarabia R. (1988). Acute epidural hematoma: an analysis of factors influencing the outcome of patients undergoing surgery in coma. J. Neurosurg..

[bib20] Maphumulo W.T., Bhengu B.R. (2019). Challenges of quality improvement in the healthcare of South Africa post-apartheid: a critical review. Curationis.

[bib21] Marmarou A., Lu J., Butcher I., McHugh G.S., Murray G.D., Steyerberg E.W. (2007). Prognostic value of the Glasgow Coma Scale and pupil reactivity in traumatic brain injury assessed pre-hospital and on enrollment: an IMPACT analysis. J. Neurotrauma.

[bib22] Peres C.M.A., Caldas J., Puglia P., de Andrade A.F., da Silva I.A.F., Teixeira M.J. (2018). Endovascular management of acute epidural hematomas: clinical experience with 80 cases. J. Neurosurg..

[bib23] Rahimi A., Corley J.A., Ammar A., Shlobin N.A., Rolle M., Mekary R.A. (2022). The unmet global burden of cranial epidural hematomas: a systematic review and meta-analysis. Clin. Neurol. Neurosurg..

[bib24] Rehman L.M.A., Khaleeq S., Zaman K. (2010). Association of Outcome of Traumatic Extradural Hematoma with Glasgow Coma Scale and Hematoma Size Mushtaq et Al. Association of Outcome of Traumatic Extradural Hematoma with Glasgow Coma Scale and Hematoma Size. Ann Pak Inst Med Sci.

[bib25] Rivas J.J., Lobato R.D., Sarabia R., Cordobés F., Cabrera A., Gomez P. (1988). Extradural hematoma: analysis of factors influencing the courses of 161 patients. Neurosurgery.

[bib26] Rohadi Muhammad Rosyidi B.P., Asra Al Fauzi, Budi Sutiono Agung (2019). Toward zero mortality in acute epidural hematoma: a review in 268 cases problems and challenges in the developing country. Interdisciplinary Neurosurgery.

[bib27] Sullivan T.P., Jarvik J.G., Cohen W.A. (1999). Follow-up of conservatively managed epidural hematomas: implications for timing of repeat CT. AJNR Am J Neuroradiol.

[bib28] Tsiouris A.J., Lui Y.W., Hodler J., Kubik-Huch R.A., Roos J.E. (2024). Diseases of the Brain, Head and Neck, Spine 2024-2027: Diagnostic Imaging.

[bib29] Yang C., Huang X., Feng J., Xie L., Hui J., Li W. (2021). Prospective Randomized Evaluation of Decompressive Ipsilateral Craniectomy for Traumatic Acute Epidural Hematoma (PREDICT-AEDH): study protocol for a randomized controlled trial. Trials.

