# Supplementary material for: Prognostic factors for early functional outcome in patients with epidural hematoma
Source: Brain Spine. 2026 Sep 5;6:106666. doi: 10.1016/j.bas.2026.106666 (PMC13594943; doi:10.1016/j.bas.2026.106666)
Supplement: Supplementary file 1 [file mmc1.docx]

| **Supplementary Table 1: Comparison of clinical, radiological, and timing characteristics between favorable and unfavorable outcomes in patients with severe TBI (n=62)** | | | | | |
| --- | --- | --- | --- | --- | --- |
| **Parameters** | **Unfavorable outcome^1^** | | **Favorable outcome^1^** | | ***p-value*** |
| **Patients (n=62)** | **18** | *(29%)* | **44** | *(71%)* |  |
| **Clinical Factors** |  |  |  |  |  |
| Median age, in years *(IQR^2^)* | 31.0 *(22.5-39)* | | *27 (23-33.8)* | | *.079* |
| Pupils equal and reactive | 8 | *(44.4%)* | 25 | *(56.8%)* | *.375* |
| Abnormal pupillary status | 10 | *(55.6%)* | 19 | *(30.6%)* |  |
| **Radiological factors^3^** | **Median** | ***IQR*** | **Median** | ***IQR*** |  |
| Midline shift (mm) | 6.8 | *(1.5-13.1)* | 8.45 | *(4.68-11.93)* | *.192* |
| Clot thickness (mm) | 26.35 | *(18-34.63)* | 26.7 | *(19-35.4)* | *.936* |
| Clot volume (cm^3^) | 86.9 | *(20.5-148.3)* | 62.7 | *(39.93-104.68)* | *.947* |
|  |  | |  | |  |
| **Time intervals (h)** | **Median** | ***IQR*** | **Median** | ***IQR*** | ***p-value*** |
| Injury-to-admission^5^ | 4 | *(2.5-10.75)* | 5 | *(2-17)* | *.775* |
| Admission-to-surgery^4,5^ | 6.5 | *(4-20.5)* | 10 | *(7-14.25)* | *.311* |
| Injury-to-surgery^4,5^ | 11 | *(8.75-34.75)* | 19 | *(13-36)* | *.122* |
| TBI = traumatic brain injury, GOSE = Glasgow outcome scale extended, IQR = Interquartile range ^1^ Unfavorable outcome (GOSE 1-5), Favorable outcome (GOSE 6-8),  ^2^ n=61; data missing in 1 patient  ^3^ Radiological data available for MLS and clot volume in 88.7%, clot thickness in 85.5%  ^4^ 54 patients in the severe TBI cohort underwent surgery  ^5^ Timing data available for injury-to-admission in 98.4%, admission-to-surgery in 96.3%, injury-to-surgery in 98.1% | | | | | |
